# VacStent as an Innovative Approach in the Treatment of Anastomotic Insufficiencies and Leakages in the Gastrointestinal Tract—Review and Outlook

**DOI:** 10.3390/life14070821

**Published:** 2024-06-27

**Authors:** Alexander Yohannes, Judith Knievel, Jonas Lange, Arno J. Dormann, Ulrich Hügle, Claus F. Eisenberger, Markus M. Heiss

**Affiliations:** 1 Center for Interdisciplinary Visceral Medicine, Department of Gastroenterology, Gastrointestinal Oncology, Gastrointestinal Infections and Nutritional Medicine, Cologne-Holweide and Merheim Medical Centers, 51109 Cologne, Germany; yohannesa@kliniken-koeln.de (A.Y.); dormanna@kliniken-koeln.de (A.J.D.); huegleu@kliniken-koeln.de (U.H.); 2 Center for Interdisciplinary Visceral Medicine, Department of Abdominal, Tumor, Transplant and Vascular Surgery, Cologne-Merheim Medical Center, Witten/Herdecke University, 51109 Cologne, Germany; knievelj@kliniken-koeln.de (J.K.); langej@kliniken-koeln.de (J.L.); eisenbergercf@kliniken-koeln.de (C.F.E.)

**Keywords:** VacStent, anastomotic insufficiency, endoscopic vacuum therapy, gastrointestinal leakage, covered stent, esosponge

## Abstract

Anastomotic insufficiencies are severe complications of abdominal surgery, often leading to prolonged hospitalization, serious tissue inflammation, and even sepsis, along with the need for recurrent surgery. Current non-surgical treatments such as self-expanding metal stents (SEMSs) and endoscopic vacuum therapy (EVT) have limitations, including stent migration or perforation. This review evaluates the effectiveness of the VacStent GI^TM^ (Möller Medical GmbH, Fulda, Germany), a novel medical device combining SEMS and negative-pressure wound therapy in treating gastrointestinal leaks. Data were gathered from four prospective studies and compared with existing treatments. Studies on the VacStent GI^TM^ application demonstrate technical success and competitive closure rates in upper gastrointestinal leaks, with minimal complications reported. Comparative analyses with SEMS and EVT reveal promising and most importantly equally good outcomes while maintaining the possibility for sustained enteral nutrition and reducing the risk of stent migration. The VacStent GI^TM^ presents a promising alternative to current non-surgical treatments. Ongoing research aims to validate its efficacy in lower gastrointestinal leaks and comprehensively establish its role in leak management. Further investigation is necessary to confirm these findings and optimize treatment protocols. Future usages of the VacStent GI^TM^ in colonic anastomotic insufficiencies promise an effective approach and might be able to lower the rates of necessary implementations of a stoma.

## 1. Introduction

Anastomotic insufficiencies and leakages are frequently seen as major complications of abdominal surgery with high mortality rates [1]. They often result in prolonged hospitalization and the severe inflammation of surrounding tissue, leading to mediastinitis and peritonitis caused by the leakage of gastrointestinal fluids [2]. It is not uncommon for patients to need surgical revision after anastomotic insufficiency to adequately treat the resulting complications [3]. For the lower gastrointestinal (GI) tract, this often leads to the creation of an anus praeter to allow the anastomosis to heal adequately. Regularly applied non-surgical treatments for the esophagus as well as the colon include the usage of a self-expanding metal stent (SEMS) and endoscopic vacuum therapy (EVT), which have been proven to treat anastomotic insufficiencies successfully [4].

Due to gastrointestinal motility, SEMSs are prone to dislocation and migration in the GI tract, making frequent corrective endoscopies necessary to ensure effective therapy [5]. Endoscopic vacuum therapy, on the other hand, which generally includes a sponge connected to a drainage tube connected to an extracorporeal vacuum pump, is proven to be effective in healing upper and lower GI leaks following an anastomotic insufficiency. Without a nasogastric feeding tube, a vacuum system solely relying on a sponge leads to a complete blockage of the lumen and therefore prevents enteral nutritional intake, which would lead to enteral mucosa atrophy [6]. Patients who are unable to swallow often experience a significant decline in their quality of life. This can lead to reduced therapy adherence and a worsening of existing psychiatric comorbidities due to the unpleasant experience of vacuum therapy and the inability to consume anything orally. These patients must continuously expel accumulated saliva.

Recent comparative studies have provided deeper insights into the efficacy of SEMS versus EVT. For instance, Schäfer’s retrospective analysis of 351 patients revealed that EVT achieved a closure rate of 89.4% compared to 69.0% for SEMS [4]. Moreover, the mortality rate due to strictures was significantly lower in the EVT group (5%) than in the SEMS group (16%). We will further deliberate on the comparison of using SEMS versus EVT later. The advantage of the VacStent GI^TM^, particularly its ability to facilitate enteral nutrition without additional feeding tubes, further underscores its clinical utility.

In 2021, Lange et al. published a study testing the applicability and effectiveness of the VacStent GI^TM^, a medical device aiming to combine the benefits of SEMS and negative-pressure wound therapy in anastomotic leakages following upper GI surgery, showing promising results [7]. This has been followed up by a multicenter prospective study in 2023 providing promising data regarding the efficacy and applicability of the VacStent GI^TM^ for upper GI leaks. A modified version of the VacStent GI^TM^ is currently being evaluated for lower gastrointestinal leakages.

## 2. Materials and Methods

**Figure 1:**
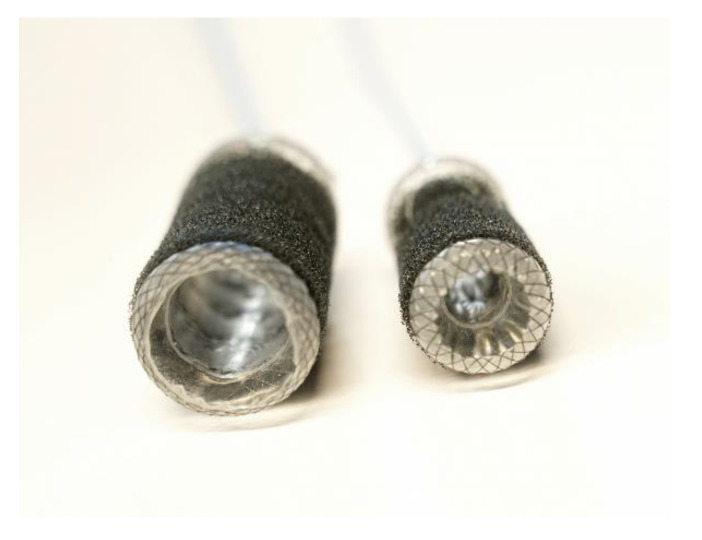
VacStent GI^TM^: **left**-side VACStent Colon (inner diameter 25 mm), **right**-side VACStent Esophagus (inner diameter 12 mm).

The VacStent GI^TM^ is a medical device manufactured by Möller Medical GmbH (Fulda, Germany) and consists of a combination of a SEMS and a cylindrical polyurethane sponge wrapped around it. Negative wound pressure is transmitted via a drainage catheter that exits the body transnasally or transanally. Before implementation, the VacStent GI^TM^ is stabilized by two additional catheters, one on the outside keeping the VacStent GI^TM^ together until it is placed, and one on the inside, enabling the correct placement of the VacStent GI^TM^. The VacStent GI^TM^ is available in various sizes depending on the application site. For use in the upper gastrointestinal tract, the VacStent GI^TM^ has a lumen of 12–14 mm, while for the lower gastrointestinal tract, it has a lumen of 25 mm, meant to ensure stool passage [8,9] (Figure 1).

For experienced endoscopists, the insertion of the VacStent GI^TM^ is not expected to be overly complicated. A guiding wire is endoscopically placed. After advancing to be sufficiently proximal to the leakage site and after the confirmation of adequate wire placement, the endoscope is withdrawn. Lubrication facilitates smooth stent insertion into the esophagus. The guiding wire is then threaded through the inner catheter’s olive hole, allowing controlled advancement of the pre-prepared stent under continuous optical guidance. Positioning ensures the proximal end of the stent lies 1 to 2 cm above the upper edge of the leakage, with the distal end positioned similarly below, verified visually before final deployment. The complete release is monitored under optical control. Post-deployment, the sponge cylinder is flushed with at least 40 mL of saline with subsequent observation for approximately 3 min to ensure optimal expansion. The introducer is then removed under optical guidance to prevent stent displacement, ensuring the drainage catheter remains in place for effective fluid drainage. Connection to a vacuum pump is established, initially set between 40 and 125 mmHg [9].

To provide an initial overview of VacStent GI^TM^ research results to date, this review summarizes the results from four prospective studies using VacStent GI^TM^ for upper gastrointestinal leaks. Studies comparing the use of conventional SEMS and EVT with an endosponge for treating gastrointestinal leaks are summarized in a review by Schäfer, which we used as a benchmark [4].

**Figure 2:**
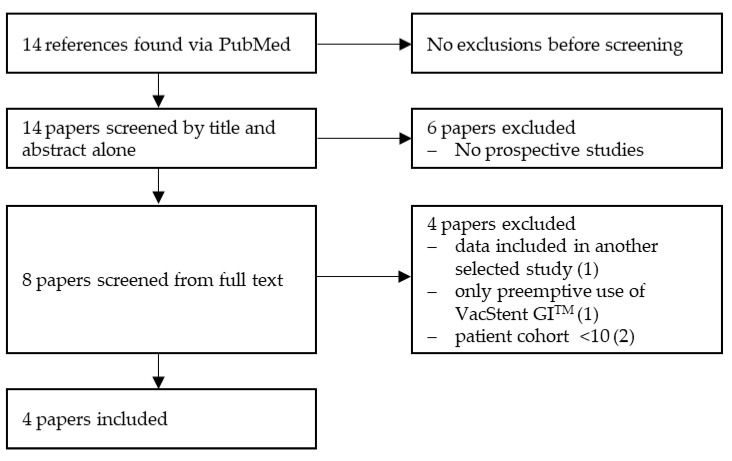
PRISMA flow diagram of identification of VacStent studies.

As a structured method of gathering the data, we conducted a PRISMA-conforming acquisition of suitable papers. We used the keyword “VACStent” and searched for current research via PubMed (Figure 2). We found 14 papers in which the VacStent GI^TM^ was mentioned. We screened these papers by title and abstract alone, which led to the exclusion of six papers because they were not prospective studies. The remaining 8 papers were screened by full text. We continued to exclude four papers because the results of one prospective study were already included in another study we continued to select, one study only highlighted the preemptive usage of the VacStent GI^TM^, and two studies had a patient cohort of fewer than 10 patients. We decided to exclude the latter studies because we wanted to include studies with a comparable patient cohort rather than studies comparing EVT with SEMS.

While there are other options for non-surgical treatment of GI leaks, such as the use of Over-the-Scope-Clips (OTSC clips) for small mucosal defects in the lower GI tract, the focus of this review will be on SEMS and EVT in comparison to the VacStent GI^TM^, as the VacStent GI^TM^ is composed of the aforementioned medical devices [10].

As mentioned above, studies describing the efficacy of treating upper GI leakages with a VacStent GI^TM^ have already been published. At our center, we are currently testing safety and efficacy for lower GI leakages. A prospective, multicenter, open-label feasibility study and a registry study are currently being conducted. For the feasibility study, patients will be eligible for inclusion if they present with a spontaneous, iatrogenic, or post-operative leakage in the lower GI tract (1) if the accessibility of the leakage with the VacStent GI^TM^ delivery system is warranted (2), if the patient 18 years or older, (3) and if they fill out a written informed consent form (4). Fixed exclusion criteria, of which there are seven, include concurrent participation in another interventional study (1), endoscopic inaccessibility of the affected segment (2), full anticoagulation with INR > 1.5 and/or PTT > 50 s or platelets < 20.000/µL (3), unstable patients with severe sepsis and necessity for immediate surgical intervention (4), ileus with persistent vomiting (5), individuals in dependency or employment relationship with the sponsor or investigator, (6) and institutionalization due to court or government order (7). As a first step, 15 patients will be included in the study to determine the feasibility and efficacy of the VacStent GI^TM^ in the lower GI tract.

We expect to have recruited the necessary patient collective by late summer 2024. Currently, we have included six patients who received treatment of anastomotic insufficiency with the VacStent GI^TM^ Colonm and a total of ten patients have received a VacStent GI^TM^ in general for postoperative lower GI leakage. Additionally, nine patients have had a VacStent GI^TM^ implemented to prevent anastomotic insufficiencies. We will give an outlook on ongoing research regarding the VacStent GI^TM^ Colon later on.

A parallel conducted registry study is recording all applications of the VacStent GI^TM^ in the upper and lower GI tract: preemptive applications, potentially high-risk anastomoses, and patients who received a VacStent GI^TM^ Esophagus following a leakage in the lower GI tract after colorectal surgery. This was necessary because the VacStent GI^TM^ Colon, which has been optimized for colorectal GI leaks, had not been made available to us for clinical use until around mid-2023. Nevertheless, the collected data will also be important for evaluating the underlying concept.

## 3. Results

In their study, Chon et al. describe the treatment of 10 patients with VacStent GI^TM^ for upper-GI tract leaks [11]. These leakages had a variety of etiologies, including iatrogenic perforation following transesophageal echocardiography, Boerhaave syndrome, esophageal fistula, and anastomotic leakage following upper GI surgery. All interventions were technically successful, with only minor complications such as incomplete stent expansion and loss of sponge during extraction. Overall, clinical success was achieved in 70% of patients without further intervention, two patients were re-treated with an Eso-SPONGE^®^ (B. Braun, Melsungen, Germany), and one patient had to undergo surgery.

Pattynama et al. described the use of VacStent GI^TM^ in a cohort of 10 patients with anastomotic leakage after esophageal resection [12]. VacStent GI^TM^ treatment was technically successful; in one patient with Boerhaave syndrome, a vacuum sponge was initially placed to drain the wound cavity. After significant shrinkage of the wound cavity, the treatment was switched to a VacStent GI^TM^. Overall, the closure of the leak was achieved in all patients.

Lange et al. summarized the results of three prospective study cohorts in Germany in which a total of 50 patients were treated with the VacStent GI^TM^ [13]. These patients had postoperative esophageal leaks or iatrogenic injuries due to various causes of anastomotic insufficiency or iatrogenic perforation (40/50). Ten patients received a VacStent GI^TM^ preemptively. Treatment with VacStent GI^TM^ usually followed previous interventions, mostly with Eso-SPONGE^®^. The application was also technically successful, and 80% of the patients showed complete healing of the leakage.

The use of the VacStent GI^TM^ in the treatment of esophageal leaks is consistent across the publications. In all reports, the placement of the VacStent GI^TM^ was generally technically successful, and in most patients (70–100%), the GI leak was completely repaired after treatment. In addition, there was no evidence of serious complications (i.e., need for surgery as a direct complication of the VacStent GI^TM^, worsening of an infection, etc.) related to VacStent GI^TM^ treatment in any of the reports. The selected studies show that the VacStent GI^TM^ can effectively treat small transmural defects on its own, while larger leakages, especially those with wound cavities, were generally pretreated with other medical devices. At this stage, no conclusions can be drawn about the efficacy of the VacStent GI^TM^ in larger defects. Further data are required.

In Schäfer’s retrospective study, he used the data from eight publications, totaling 351 patients. Within this created cohort, 157 patients received treatment via EVT for upper GI leakages, constituting 44.7% of the total population, while the remaining 194 patients were allocated to the cSEMS group, representing 55.3% of the total population. The gender distribution varied between the groups, with 78% male and 22% female patients in the cSEMS group and 86% male and 14% female patients in the EVT group [4].

Regarding treatment outcomes, the closure rate was notably higher in the EVT group, with 89.4% (126/141 patients) achieving closure, compared to 69.0% (126/182 patients) in the SEMS group. Treatment switches occurred in both groups, with ten patients transitioning from EVT to SEMS due to minimal findings or cSEMS-treatable strictures and 15 patients switching from SEMS to EVT treatment. Surgical revisions were performed on 14 patients in the EVT group and 18 patients in the cSEMS group. The mortality rate due to strictures was 5% (5 out of 97 patients) in the EVT group and 16% (21 out of 129 patients) in the cSEMS group [4].

Comparing the success of the respective medical devices in terms of the closure of a gastrointestinal leak, we found no disadvantage in the use of the VacStent GI^TM^ in the upper gastrointestinal tract compared to the already-established SEMS or therapy with EVT. The advantages of the VacStent GI^TM^, namely the uncomplicated possibility of enteral nutrition and therefore the lack of a need for an additional nasogastric feeding tube, still stand out. We believe that this will also bring significant benefits to the patient and facilitate compliance. Stent migration, a common complication of SEMS, is very rare due to the continuous suction against the esophageal wall [10].

When comparing the included prospective studies, we first examined the different timing of the VacStent GI^TM^ application. We noted that the VacStent GI^TM^ was used with varying frequency (22.5–85%) as a first-line treatment [Table 1]. This can be possibly explained by potential limitations such as a too-large wound cavity and its intended reduction by intracavitary sponge placement before VacStent GI^TM^ implantation. In cases where the VacStent GI^TM^ was used as a first-line treatment, we observed successful leakage treatment in 70.6–100% of cases [Table 1]. The data from the largest prospective study published by Lange et al. in 2023 do not provide information on the successful closure of the VacStent GI^TM^ as a first-line treatment for leakage [10]. Since this study has been conducted at our center, we were able to pool the data from the primary source. We have seen a successful closure of the leakage in eight of nine cases. Surgery was necessary due to insufficient closure of the leakage in one case. Pooling the data, we see an overall successful treatment in terms of the absence of detectable anastomotic insufficiency in 27 out of 34 patients (79%) [Table 2]. In cases where the VacStent GI^TM^ was used as a stand-alone device, we see 27 out of 29 (93%) successful treatments of anastomotic insufficiency [Table 2]. In four cases, the VacStent GI^TM^ treatment was followed by a single EVT treatment because of insufficient closure of the anastomotic insufficiency, and in one case, surgical treatment of the anastomotic insufficiency had to be performed. Looking at the timing of VacStent GI^TM^ deployment and the overall success rate of the non-surgical treatments, we see a success rate of 68 out of 80 patients (85%). This includes the deployment of the VacStent GI^TM^ at any stage of the non-surgical treatment of the leak. SEMS or EVT may be used before or after the application of the VacStent GI^TM^. Like Schäfer’s publication, where 89% of the studies described EVT and 69% of the studies described SEMS being successful, we see similarly successful results here [4]. Therefore, the use of VacStent GI^TM^ therapy does not appear to be inferior in the non-surgical treatment of anastomotic insufficiency.

On a side note, we want to mention that we are currently recruiting patients in our prospective study regarding the applicability of the VacStent GI^TM^ in the lower GI tract, so conclusive results cannot be given at this point. These further patients have received a preemptive VacStent GI^TM^ Colon placement in a high-risk anastomosis setting immediately post-surgery similar to the preemptive placement in the upper GI tract. We have not seen any post-operative anastomotic insufficiency to date. This also correlates with the published results regarding preemptive use in the upper GI tract [15]. In the lower GI tract, the VacStent GI^TM^ remained in place for seven days before being removed without complications. Fortunately, we were able to avoid the need for a protective ileostomy in every case. There have been no major complications to date.

We look forward to further results that will provide a reliable statement on the safety and applicability of the VacStent GI^TM^ in the preemptive setting of high-risk lower GI anastomoses.

## 4. Discussion

The current dataset suggests that the VacStent GI^TM^ presents a promising and substantiated alternative to conventional treatment modalities such as SEMS or EVT. Of notable significance is the potential for sustained enteral feeding with a markedly reduced rate of stent migration in comparison to SEMS, as delineated in the pilot study, which serves to bolster its credibility [7]. Additionally, the induction of granulation tissue attributed to continuous negative pressure provides further support for the adoption of the VacStent GI^TM^. Prior investigations have depicted the VacStent GI^TM^ as a versatile adjunct in the management of leaks alongside SEMS or EVT. However, a conspicuous absence in the literature pertains to the rationale underlying the preference for an alternative medical device in described clinical scenarios.

The comprehensive assessment of the VacStent GI^TM^ utility in treating small to medium-sized leaks in the upper gastrointestinal tract throughout the entire healing process remains a subject of ongoing inquiry. This underscores the imperative for a larger-scale investigation involving a larger cohort to establish definitive conclusions. Nevertheless, existing research attests to the conceptual viability, safety profile, and broad applicability of the VacStent GI^TM^. We believe that the prolonged utilization of the VacStent GI^TM^ may yield superior outcomes to those observed in initial clinical trials. Presently, our institution employs a standardized protocol of seven-day VacStent GI^TM^ placement duration for both upper and lower GI tract cases.

Furthermore, Chon et al. delineate a setback concerning enteral nutrition administration via high-caloric protein beverages [14]. The absence of corroboration precludes the definitive validation of these findings, thereby warranting further scientific scrutiny. This represents a compelling avenue for subsequent exploration. Drawing upon our clinical experience with the VacStent GI^TM^ in both esophageal and colonic leakages, we find it suitable for oral intake of liquid or meshed food without complications in either anatomical site.

Considering previously published studies reporting on the success of SEMS and EVT, it is noticeable that similar-sized study populations are generally observed. For instance, in Schäfer’s compilation, it is noted that between 10 and 102 patients (median: 24.5 patients) were included in the studies [4]. Certainly, there are additional study findings on this subject matter that could be included in a larger-scale review comparing SEMS with EVT; however, we nonetheless observe fundamentally comparable study populations. In the VacStent GI^TM^ studies, a median patient cohort of 15 patients (range: 10–40) is evident, thus indicating comparability. Across the VacStent GI^TM^ studies, an overall achieved occlusion rate of 85% can observed if the usage of other non-surgical treatment approaches mentioned above is included. When the use of the VacStent GI^TM^ as a stand-alone device is compared with an integrated approach including the use of other non-surgical leakage treatments, the results appear to be even better. The VacStent GI^TM^ is therefore not only a real alternative to the singular treatment of anastomotic insufficiencies and gastrointestinal leaks in general but can also be integrated as an adjunct to the previously rather narrow range of non-surgical options for defect treatment. The experience of Lange et al. with the VacStent GI^TM^ appears to be particularly encouraging in terms of preventing or limiting septic disease [7]. Necrotic cavities > 2 cm in diameter are a limiting factor for VacStent GI^TM^ placement [14]. In these cases, a defect healed after the use of an intracavitary vacuum sponge with additional intraluminal vacuum sponge therapy.

The experience gained in the upper GI tract is naturally useful for the planned use in the colon. The colonic environment is more challenging due to the continuous peristalsis, the larger lumen, and the consequent need for a larger lumen stent. The previously described conflicting experiences regarding the possibility of enteral feeding will be of greater importance in assessing the applicability of the VacStent GI^TM^ Colon, as any obstruction of the stent lumen by food components will naturally lead to clinical symptoms after a latency period.

An iatrogenic obstruction with the potential for causing a mechanical ileus could lead to an avoidable deterioration of the patient’s overall condition but can generally be managed by stent replacement, as has been reported in cases of colonic SEMS obstruction [16]. However, in the study by Chon et al., stent occlusion did not seem to have happened. Rather, the VacStent GI^TM^ was shown to insufficiently generate negative pressure [14]. It is not described at what point in time these complications occurred, but a possible solution might be to choose a different liquid nutrition or to change the intervals at which the VacStent GI^TM^ is changed. In contrast, the liquid enteral nutrition described elsewhere appeared to be possible without complications [17].

A further observation that speaks in favor of the feasibility of the VacStent GI^TM^ for anastomotic insufficiencies is that no serious adverse device effects (SADEs) were described. In the studies presented, the VacStent GI^TM^ is operated with a negative pressure of up to 125 mmHg [7,14]. Despite the different configuration of a traditional EVT using a vacuum sponge, this does not appear to promote SADEs. In contrast to vacuum sponge therapy, the generated negative pressure presses a significantly less elastic nitinol stent against the wall of the esophagus compared to a sponge. At our center, we also use a negative pressure of 125 mmHg in both the esophagus and the colon. We have seen good results with this approach. No increased incidence of SADEs has been observed so far.

In this review, the data compiled and publications about the utilization of the VacStent GI^TM^ in a preemptive setting were not included. Nonetheless, it is important to acknowledge the potential of preemptive, perioperative implementation of the VacStent GI^TM^, particularly in clinical settings characterized by a heightened risk of anastomotic insufficiency. A study by Lange et al. has tested the feasibility and successful application of the VacStent GI^TM^ in a preemptive setting [15]. The study encompassed a cohort of nine patients who underwent Ivor Lewis esophagectomy with end-to-side esophagogastrectomy after neoadjuvant chemotherapy. The findings, akin to previously published data that have been subject to comparison and analysis in this review, underscore an overall technical success, reaffirming the intuitive utility of the VacStent GI^TM^ for interventional endoscopists. Notably, the patients in the study were able to resume oral intake of fluids on the first postoperative day. Additionally, they received prophylactic periinterventional antibiotic therapy and underwent regular removal of the VacStent GI^TM^, with the potential for reinsertion of a new device after 5–7 days, as necessitated. Encouragingly, the data indicate that eight out of the nine patients required only one VacStent GI^TM^ and did not encounter postoperative anastomotic insufficiency. However, one patient exhibited signs of esophageal leakage 10 days after surgery, a complication that was effectively managed with two consecutive applications of the VacStent GI^TM^. This particular case serves as an additional indicator not only for the preventative efficacy of the VacStent GI^TM^ but also for its therapeutic potential in addressing anastomotic leakages.

In the selection of a preferred non-surgical method for the treatment of anastomotic leakages, the aspect of cost-effectiveness gains more significance. If multiple equally effective devices are available on the market, it is prudent to prioritize the option that entails the least consumption of resources and financial investment. For instance, the VacStent GI^TM^ necessitates removal and potential replacement after approximately 7 days, while alternative EVT devices such as the Eso-SPONGE^®^ require replacement every 2–5 days based on our experience. The replacement process involves the allocation of personnel resources including physicians, nurses, and endoscope reprocessing technicians, as well as incurring material costs for disinfection and sterilization equipment, personal protective gear, protective sheaths, and disposable valves.

It is therefore sensible that opting for an alternative device that requires fewer replacements not only streamlines management for hospitals but also presents as a more cost-effective option overall. Furthermore, the environmental implications of such a choice cannot be overlooked, as it is deemed to be more environmentally sustainable. Therefore, in the interest of operational efficiency, financial prudence, and environmental responsibility, the selection of a medical device with reduced replacement frequency warrants careful consideration.

## 5. Conclusions

The evaluation of the VacStent GI^TM^ as an innovative approach to the treatment of anastomotic insufficiencies in the gastrointestinal tract shows promising results. The integration of SEMS with EVT addresses its limitations. The studies demonstrate competitive closure rates with no reported SADEs in the upper gastrointestinal leaks.

While the VacStent GI^TM^ shows promise, ongoing research efforts aim to validate its efficacy in lower gastrointestinal leaks and optimize treatment protocols. Challenges such as insufficient negative pressure generation and potential obstruction from food components in the colonic environment require further investigation. Nevertheless, the experience gained in upper GI applications offers valuable insights for future colonic applications.

Overall, the VacStent GI^TM^ represents a compelling alternative for the treatment of gastrointestinal leaks, with potential benefits in terms of patient outcomes and treatment efficacy. Further investigation and refinement of its utility hold promise for improving clinical practice and patient care in the management of anastomotic insufficiencies throughout the gastrointestinal tract. Further studies are warranted to substantiate these findings and elucidate the full scope of its clinical applicability.

## Figures and Tables

**Table 1:** Comparison of the success rates of the VacStent GI^TM^ as a first-line treatment.

Source	First-Line Treatment with the VacStent GI^TM^ (Percentage of Cohort)	Closure of Leakage Achieved after Primary Use of the VacStent GI^TM^	EVT Used after VacStent GI^TM^ Application within This Cohort	SEMS Used after VacStent GI^TM^ Application within This Cohort
Lange et al. [10]	9/40 (23%)	8/9 * (89%)	0	2 *
Chon et al. [12]	5/10 (50%)	4/5 (80%)	0	0
Pattynama et al. [13]	3/10 (30%)	3/3 (100%)	0	0
Chon et al. [14]	17/20 (85%)	12/17 (71%)	4	0
Combined	34/80 (42.5%)	27/34 (79%)	-	-

* data received from primary source.

**Table 2:** Application of the VacStent GI^TM^ as the sole treatment of anastomotic leakage compared to the usage with necessary adjunctive therapies.

Source	Patients with Upper GI Leakage	Age	Sexm/f	Patients with AI	Sole Use of VacStent GI^TM^	Sole Use of VacStent GI^TM^ with Consecutive Closure of the Leakage	Closure of Leakage Achieved Including Usage of Additional Non-Surgical Treatment	Usage of EVT before/after VacStent GI^TM^ Treatment	Usage of SEMS before/after VacStent GI^TM^ Treatment	Reported SADE
Lange et al. [10]	40	64 (23–92)	35/15	26	9	8/9 (89%) *	32/40 (80%)	24/0	4/2 *	0
Chon et al. [12]	10	63 (28–84)	8/2	4	5	4/5 (80%)	9	0/2	1/0	0
Pattynama et al. [13]	10	65 (62–73)	10/0	8	3	3/3 (100%)	10	7/0	0/0	0
Chon et al. [14]	20	61	20/0	18	12	12/12 (100%)	19/20	3/7	2/0	0
Combined	80	-	68/17	56	29	27/29 (93%)	68/80 (85%)	30/9	7/2	0

* data received from primary source.

## Data Availability

No new data were created or analyzed in this study. Data sharing is not applicable to this article.
